# Modulation of the Receptor Tyrosine Kinase TIE2/*Tek* Pathway by NRF2 Activation in Neurovascular Endothelial Cells

**DOI:** 10.3390/ijms27020770

**Published:** 2026-01-13

**Authors:** Eduardo Cazalla, Ángel Juan García-Yagüe, Marta Pajares, José Jiménez-Villegas, Maribel Escoll, Ana I. Rojo, Antonio Cuadrado

**Affiliations:** 1 Department of Biochemistry, School of Medicine, Autonomous University of Madrid (UAM), 28049 Madrid, Spain; 2 Instituto de Investigaciones Biomédicas “Sols-Morreale” (CSIC-UAM), 28029 Madrid, Spain; 3 Instituto de Investigación Sanitaria La Paz (IdiPaz), 28046 Madrid, Spain; 4 Centro de Investigación Biomédica en Red de Enfermedades Neurodegenerativas (CIBERNED), 28046 Madrid, Spain; 5 Group of Neurodegenerative Diseases, Hospital Universitario 12 de Octubre Research Institute (imas12), 28041 Madrid, Spain

**Keywords:** NRF2, TIE2/*Tek*, BACH1, angiogenesis, neuroendothelium

## Abstract

The transcription factor NRF2 orchestrates diverse cellular homeostatic networks, but its role in angiogenesis remains poorly understood. Genetic and pharmacological modulation of NRF2 in mouse neuroendothelial cells altered the expression of several genes involved in endothelial biology. Among these, the TIE2/*Tek* receptor, essential for vascular development and integrity, was downregulated upon NRF2 activation, accompanied by changes in adherens and tight junction gene expression. Hemin treatment and knockdown revealed that TIE2/*Tek* repression is independent of the NRF2 repressor BACH1. mRNA stability and ChIP analyses indicated no post-transcriptional or direct transcriptional repression by NRF2. These findings suggest an alternative NRF2-dependent mechanism affecting TIE2/*Tek* levels and potentially influencing angiogenic regulation.

## 1. Introduction

The blood–brain barrier (BBB) is a complex and selective barrier that maintains the homeostasis of the central nervous system (CNS) and is essential for proper neuronal function [1]. The main constituents of the BBB are highly specialized endothelial cells (ECs), which set up a tightly sealed cellular layer that restricts molecular and cellular trafficking from the blood into the CNS. ECs in the BBB are accompanied by astrocyte end-feet projections, pericytes, and neurons. These cells, collectively known as the neurovascular unit (NVU), interact with one another and dynamically modulate neurovascular coupling, microvascular permeability, matrix interactions, neurotransmitter inactivation, neurotrophic coupling, or angiogenesis, thereby allowing adequate function of the CNS [2,3]. Most of these functions are achieved through paracrine secretion of soluble ligands, such as WNTs, VEGF, or angiopoietins (ANGPT) [4,5,6].

The ANGPT/TIE2 axis initiates signaling pathways that modulate vascular stability and angiogenesis and plays an important role in a variety of physiological and pathological processes, including inflammation, wound healing, and cancer, by regulating endothelial cell proliferation, survival, migration, invasion, and/or differentiation [7]. Downstream pathways activated by TIE2 receptors can stabilize the endothelium and reduce the loss of pericytes, thus contributing to maintaining the physiological function of the vascular system in NVU [8]. Disruption of the TIE2 receptor can lead to endothelial cell activation or dysfunction, which can contribute to the pathogenesis of NVU [7,9]. Although the mechanisms have not been fully elucidated, ANGPT/TIE2 axis malfunction results in altered endothelial cell activation, neuroinflammation responses, neovascularization, and modified vascular permeability [9].

In this context, the transcription factor NRF2 (nuclear factor (erythroid-derived 2)-like 2) coordinates a wide network of transcriptional responses directed towards the preservation of cellular homeostasis. NRF2 regulates the basal and stress-inducible expression of over 250 genes that share in common a cis-acting enhancer termed antioxidant response element (ARE) and participate in several cytoprotective functions reviewed elsewhere [10]. Although some reports point to a preserving role of NRF2 in the integrity of the NVU [11,12,13,14], the molecular mechanisms involved remain poorly explored. In this study, we show for the first time a functional connection between NRF2 and the TIE2 receptor, through a mechanism that currently remains unknown. This novel axis may have an impact on endothelial and specifically BBB biology.

## 2. Results

### 2.1. Sulforaphane Reduces TIE2/Tek Expression

**Figure 1:**
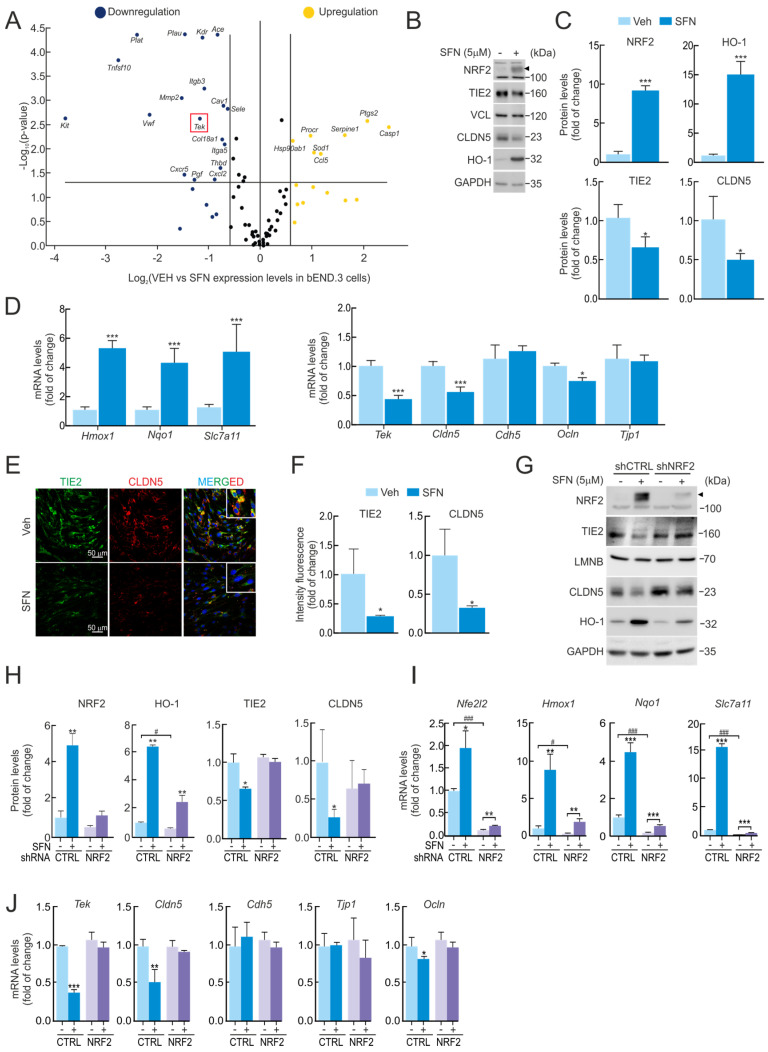
Reduced TIE2/*Tek* levels with SFN are dependent on NRF2 activity. (**A**) bEnd.3 cells were maintained under low-serum conditions (16 h, 1% FBS) and subjected to SFN (5 µM, 16 h). Expression levels of 84 endothelial genes were analyzed by quantitative real-time PCR (qRT-PCR) and normalized to the geometric mean of *Actb*, *Gapdh*, *B2m*, *Gusb*, and *Hsp90ab1* levels. Volcano plot comparing gene expression of vehicle- versus SFN-treated cells. A fold of change greater than 1.5 is represented by yellow (increased expression when compared to vehicle-treated cells) and blue (decreased expression when compared to vehicle-treated cells) dots. Data are the mean of n = 4. Statistical analysis was performed with the GeneGlobe Data Analysis Center from Qiagen. A *p*-value < than 0.05 was considered significant and is represented by a line. A red square highlights the *Tek* gene. (**B**) Representative immunoblots of NRF2 (arrowhead), TIE2, CLDN5, HO-1, and VCL, and GAPDH as a loading control from bEnd.3 cells maintained under low-serum conditions (16 h, 1% FBS) and treated with SFN (5 µM, 16 h). (**C**) Densitometric quantification of NRF2, HO-1, TIE2, and CLDN5 protein levels from representative immunoblots of B expressed as a ratio of VCL and GAPDH, respectively. Data are mean ± S.D. (n = 3). * *p* < 0.05 and *** *p* < 0.001 vs. vehicle according to Student’s *t*-test. (**D**) bEnd.3 cells were maintained under low-serum conditions (16 h, 1% FBS) and subjected to SFN (5 µM, 16 h). Transcript levels of *Hmox1*, *Nqo1*, *Slc7a11*, *Tek*, *Cldn5*, *Cdh5*, *Ocln*, and *Tjp1* were determined by qRT-PCR and normalized to the geometric mean of the levels of *Gapdh*, *Tbp*, and *Actb*. Data are mean ± S.D. (n = 3). * *p* < 0.05 and *** *p* < 0.001 vs. vehicle according to Student’s *t*-test. (**E**) Confocal analysis of double immunofluorescence with anti-TIE2 (green) and anti-CLDN5 (red) antibodies in vehicle (Veh.) or SFN-treated bEnd.3 cells. (**F**) Quantification of TIE2 and CLDN5 intensity signals in E. Values are mean ± SD (n = 3, with >50 cells counted per field). * *p* < 0.05 vs. VEH-treated cells according to Student’s *t*-test. (**G**) bEnd.3 cells were transduced with lentiviral vectors carrying short hairpin RNA against NRF2 (shNRF2) or a scramble sequence (shCTRL). Five days post-transduction, cells were maintained under low-serum conditions (16 h, 1% FBS) and subjected to SFN (5 µM, 16 h). Representative immunoblots of NRF2 (arrowhead), TIE2, CLDN5, HO-1, and LMNB, and GAPDH as a loading control. (**H**) Densitometric quantification of NRF2, HO-1, TIE2, and CLDN5 protein levels from representative immunoblots of G expressed as a ratio of LMNB and GAPDH, respectively. Data are mean ± S.D. (n = 3). * *p* < 0.05 and ** *p* < 0.01 vs. vehicle, or # *p* < 0.05 vs. shCTRL according to Student’s *t*-test. (**I**) Transcript levels of *Nfe2l2*, *Hmox1*, *Nqo1*, and *Slc7a11* and (**J**) of *Tek*, *Cldn5*, *Cdh5*, *Ocln*, and *Tjp1* from bEnd.3 cells transduced with shNRF2 or shCTRL maintained under low-serum conditions (16 h, 1% FBS) and subjected to SFN (5 µM, 16 h) were determined by qRT-PCR and normalized to the geometric mean of the levels of *Gapdh*, *Tbp*, and *Actb*. Data are mean ± S.D. (n = 3). * *p* < 0.05, ** *p* < 0.01 and *** *p* < 0.001 vs. vehicle, or # *p* < 0.05 and ### *p* < 0.001 vs. shCTRL according to Student’s *t*-test.

To understand the impact of NRF2 on endothelial cell biology, we first employed a well-characterized NRF2 pharmacological activator, SFN (5 µM, 16 h), on murine brain endothelial cells (bEnd.3). Gene expression was analyzed by employing an array containing a battery of genes involved in different key pathways for endothelial cell biology, including angiogenesis, vasoconstriction and vasodilation, inflammatory response, apoptosis, cell adhesion, coagulation, and platelet activation. As shown in Figure 1A, NRF2 induction by SFN mediated the upregulation of *Ccl5* (RANTES), *Ptgs2* (COX2 or cyclooxygenase 2), *Casp1* (caspase 1), *Hsp90ab1* (heat shock protein 90 alpha family class B member 1), *Procr* (endothelial protein C receptor), *Serpine1* (plasminogen activator inhibitor 1), and *Sod1* (superoxide dismutase 1) expression levels. To our surprise, SFN resulted in decreased expression of several genes, namely *Ace* (angiotensin-converting enzyme), *Sele* (LECAM2), *Cxcr5* (C-X-C motif chemokine receptor 5), *Col18a1* (collagen type XVIII alpha 1 chain), *Itga5* (integrin subunit alpha 5), *Cav1* (caveolin 1), *Pgf* (placental growth factor), *Plau* (plasminogen activator), *Thbd* (thrombomodulin), *Tnfsf10* (TRAIL), and *Tek* (*TEK* receptor tyrosine kinase or TIE2). We had previously noted that the TIE2 receptor was downregulated by SFN through NRF2 activation, as both proteins elicit similar protection mechanisms related to anti-inflammatory activity, cell survival, and the maintenance of the physiological function of the vascular system in NVU [1,8]. Building on this, we wanted to go further and decipher the functional relationship between NRF2 and the TIE2 receptor. To this end, we conducted experiments employing the bEnd.3 cell lines, subjected to SFN (5 µM, 16 h), and analyzed protein and mRNA levels. As expected, SFN treatment led to an increase in NRF2 protein levels and one of the well-known antioxidant target proteins, HO-1 (Figure 1B,C). Additionally, we detected elevated expression levels of its targets *Hmox1*, *Nqo1*, and the endothelial *Slc7a11* (Figure 1D). By contrast, SFN treatment resulted in a significant decrease in TIE2/*Tek* receptor protein and mRNA levels, as well as one of the tight junction (TJ) protein levels, CLDN5/*Cldn5* (Figure 1D). We also corroborated this phenomenon by employing an immunocytofluorescence assay in bEnd.3, showing a correlative decrease in both proteins upon treatment with SFN (5 µM, 16 h) (Figure 1E,F). On the other hand, we observed a significant reduction in other mRNA levels related to TJ genes, such as *Ocln*, but not *Cdh5* and *Tjp1* (Figure 1D).

Of note, these results were reproduced in human endothelial cell lines (hCMEC/D3) (Supplementary Figure S1A–C) and mouse lung endothelial cells (1g11) (Supplementary Figure S1D–F). Both endothelial cell lines were exposed to a similar treatment with SFN (5 µM, 16 h), resulting in correlative TIE2/*Tek* depletion protein and mRNA levels, thus validating that the effect of NRF2 activation can trigger a reduction in this receptor in other endothelial cells.

### 2.2. SFN-Mediated TIE2/Tek Modulation Is NRF2-Dependent

To decipher if NRF2 mediates the effect of SFN on TIE2/*Tek* reduction. bEnd.3 cells were transduced with lentiviral vectors containing shRNA against NRF2 (shNRF2) or a random sequence (shCTRL). Five days post-transduction, the cells were treated with SFN (5 µM, 16 h). As observed in Figure 1G–I, efficient NRF2 silencing resulted in decreased expression of its targets *Nfe2l2*, *Hmox1*, *Nqo1*, and *Slc7a11* both at the messenger and protein levels. SFN led to increased expression of these genes, but this response was greatly impaired in the absence of NRF2. While the absence of NRF2 did not affect the basal expression of *Tek* or its protein TIE2 (Figure 1G–J), its reduction upon SFN was abolished in shNRF2-transduced cells, pointing to NRF2 as the mediator of SFN effects on TIE2/*Tek*. Additionally, CLDN5/*Cldn5* expression was not downregulated by SFN in the absence of NRF2, showed a correlated effect with TIE2 depletion in the NRF2 activation condition, and indicated NRF2-dependent mechanisms of regulation. We also detected a significant reduction in other mRNA levels related to TJ genes, such as *Ocln*, but not *Cdh5* and *Tjp1* (Figure 1J).

We further employed an array analysis for gene expression evaluation in bEnd.3 cells transduced with shNRF2 lentivirus, which showed diminished expression of *F2r* (coagulation factor II thrombin receptor), *Pf4* (platelet factor 4), and *Pdgfra* (platelet-derived growth factor receptor alpha), and increased levels of *Itgb3* (integrin subunit beta 3), *Vcam1* (vascular cell adhesion molecule 1), *Il7* (interleukin 7), Ccl5 (RANTES), *Ccl2* (CC Motif Chemokine Ligand 2), *Cradd* (CASP2 and RIPK1 Domain Containing Adaptor With Death Domain), and *Tnfsf1o* (TRAIL) when compared to shCTRL-transduced cells (Supplementary Figure S2A). Certain changes in gene expression were observed in shNRF2-transduced cells upon SFN treatment, indicating the existence of NRF2-independent mechanisms (Supplementary Figure S2B). In both arrays, we were not capable of detecting any changes in the *Tek* gene levels.

In summary, these results indicate that pharmacological activation of NRF2 by SFN modulates the expression of several BBB-related genes, including a reduction in TIE2/*Tek* levels in an NRF2-dependent manner, as summarized in Supplementary Figure S2C. Based on these findings, we propose that changes in NRF2 levels may alter endothelial cell junctional integrity and potentially modify the structure of the NVU.

### 2.3. Genetic Overexpression of NRF2 Decreases TIE2/Tek Levels

**Figure 2:**
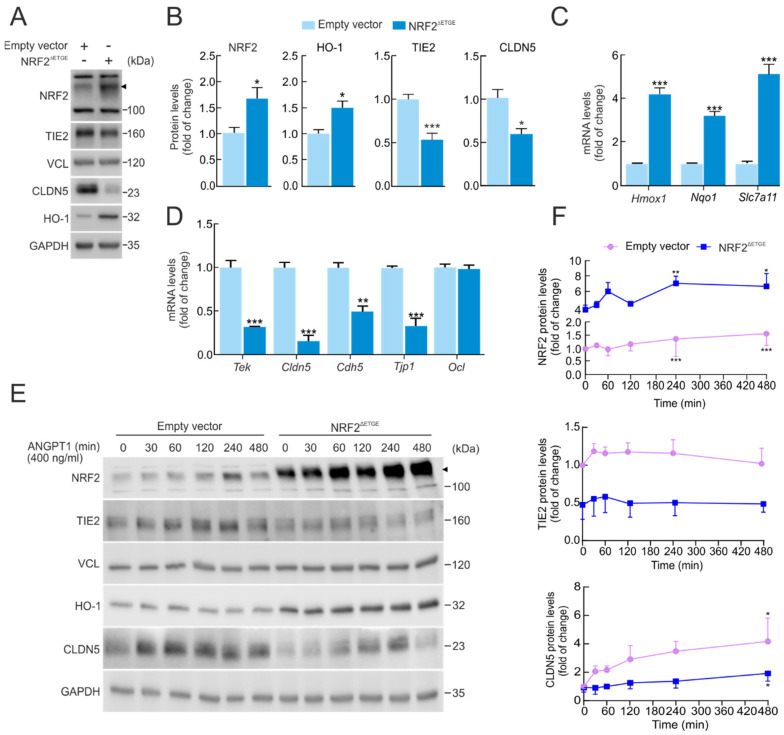
Overexpression of NRF2 alters TIE2/*Tek* levels. (**A**) bEnd.3 cells were transduced with lentiviral vectors carrying overexpressed NRF2 insensitive to KEAP1 degradation (NRF2^∆ETGE^) or a lentivirus empty vector. Five days post-transduction, cells were maintained under low-serum conditions (16 h, 1% FBS). Representative immunoblots of NRF2 (arrowhead), TIE2, CLDN5, HO-1, and VCL, and GAPDH as a loading control. (**B**) Densitometric quantification of NRF2, HO-1, TIE2, and CLDN5 protein levels from representative immunoblots of A expressed as a ratio of VCL and GAPDH, respectively. Data are mean ± S.D. (n = 3). * *p* < 0.05 and *** *p* < 0.001 vs. empty vector according to Student’s *t*-test. (**C**) Transcript levels of *Hmox1*, *Nqo1*, and *Slc7a11* and (**D**) of *Tek*, *Cldn5*, *Cdh5*, *Ocln*, and *Tjp1* from bEnd.3 cells transduced with NRF2^∆ETGE^ or empty vector maintained under low-serum conditions (16 h, 1% FBS) were determined by qRT-PCR and normalized to the geometric mean of the levels of *Gapdh*, *Tbp*, and *Actb*. Data are mean ± S.D. (n = 3). ** *p* < 0.01 and *** *p* < 0.001 vs. empty vector according to Student’s *t*-test. (**E**) bEnd.3 cells were transduced with lentiviral vectors NRF2^∆ETGE^ or an empty vector. Five days post-transduction, cells were maintained under low-serum conditions (16 h, 1% FBS) and were subjected to ANGPT1 (400 ng/mL) purified from supernatant HEK293T-stable expression (CMP-ANGPT1) to the indicated time points. Representative immunoblots of NRF2 (arrowhead), TIE2, CLDN5, HO-1, and VCL, and GAPDH as a loading control. (**F**) Densitometric quantification of NRF2, TIE2, and CLDN5 protein levels from representative immunoblots of E expressed as a ratio of VCL and GAPDH, respectively. Data are mean ± S.D. (n = 3). * *p* < 0.05, ** *p* < 0.01 and *** *p* < 0.001 vs. time 0 according to a one-way ANOVA followed by a Bonferroni post hoc test.

To gain deeper insight into the impact of NRF2 on the drop in TIE2/*Tek* levels, we performed a genetically manipulated NRF2 activity. For this purpose, we transduced bEnd.3 with a lentivirus expressing a constitutive active form of NRF2 (NRF2^ΔETGE^) or pLENTI-PURO empty vector as a control for five days. The construction NRF2^ΔETGE^ mutant is resistant to KEAP1-mediated proteasomal degradation, as it lacks the specific affinity motif to promote degradation through the ubiquitin–proteasome system [15]. Overexpression of active NRF2 was confirmed by an increase in NRF2 and HO-1 levels by immunoblot (Figure 2A,B). Consistently, mRNA levels of NRF2-target genes, namely *Hmox1*, *Nqo1*, and the endothelial *Slc7a11*, were also increased (Figure 2C). Conversely, NRF2 overexpression led to reduced TIE2/*Tek* protein and mRNA levels, as well as tight junction CLDN5/*Cldn5*, as shown in Figure 2A–D, supporting a role of NRF2 in the regulation of TIE2 and its targets CLDN5. Additionally, NRF2^ΔETGE^ led to a significant reduction in other mRNA levels related to TJ genes, such as *Cdh5* and *Tjp1*, but not *Ocln* (Figure 2D).

We further sought to determine the impact of NRF2^ΔETGE^ overexpression on the TIE2 receptor pathway, particularly in response to its principal ligand ANGPT1. To this end, we generated and purified the recombinant ligand CMP-ANGPT1 through stable expression in HEK293T cells (see Section 4, Materials and Methods). As shown in Figure 2E,F, lentiviral-mediated overexpression of NRF2^ΔETGE^ markedly decreased TIE2 protein levels compared with the empty vector control. This reduction was accompanied by a significant impairment in TJ protein CLDN5 induction following CMP-ANGPT1 (400 ng/mL) stimulation over time. Interestingly, activation of TIE2 receptor signaling by CMP-ANGPT1 slightly increased NRF2 protein levels, even in the absence of the KEAP1-modulated DETGE domain, suggesting that downstream AKT/GSK-3 signaling may contribute to NRF2 stabilization [16].

By contrast, activation of TIE2 signaling by CMP-ANGPT1 under NRF2 genetic ablation conditions did not produce any significant change in CLDN5 protein levels, although a modest increase in TIE2 protein abundance was detected (Supplementary Figure S3A,B).

These results suggest that NRF2 modulates TIE2 signal transduction and endothelial TJ integrity, indicating a reciprocal regulatory relationship in which NRF2 activation dampens TIE2 responsiveness, thereby influencing neurovascular homeostasis.

### 2.4. BACH1 Does Not Repress TIE2/Tek Expression Levels

Pharmacological or genetic manipulation of NRF2 in brain endothelial bEnd.3 cells resulted in a suppressed expression of several BBB-related genes, including *Tek*. Although a direct transcriptional repressor role for NRF2 has been recently proposed [17], our preliminary analysis did not reveal NRF2 binding to the TIE2/*Tek* promoter within the RPA1 sequence. Therefore, we searched for potential transcriptional repressors that could mediate NRF2-dependent downregulation of TIE2/*Tek*. We initially focused on BACH1, a well-known NRF2 competitor that represses ARE-containing genes through MAREs (MAF Recognition Elements) [18]. To this end, we analyzed available chromatin immunoprecipitation (ChIP) datasets for BACH1 and BACH1-binding partners MAFF, MAFK, and MAFG, which are MARE-binding transcription factors [19]. Subsequently, we employed a Python 3.4 program-based in silico pipeline [20] to screen the TIE2/*Tek* promoter region for consensus MARE motifs defined in the JASPAR database [21], considering sequences with a relative score above 90%. This analysis identified a single putative MARE site within the TIE2/*Tek* promoter, corresponding to the sequence 5′-CCTGACTCAGCT-3′ (genomic coordinates mm10 chr4:94738039-94738051; relative score = 0.916).

**Figure 3:**
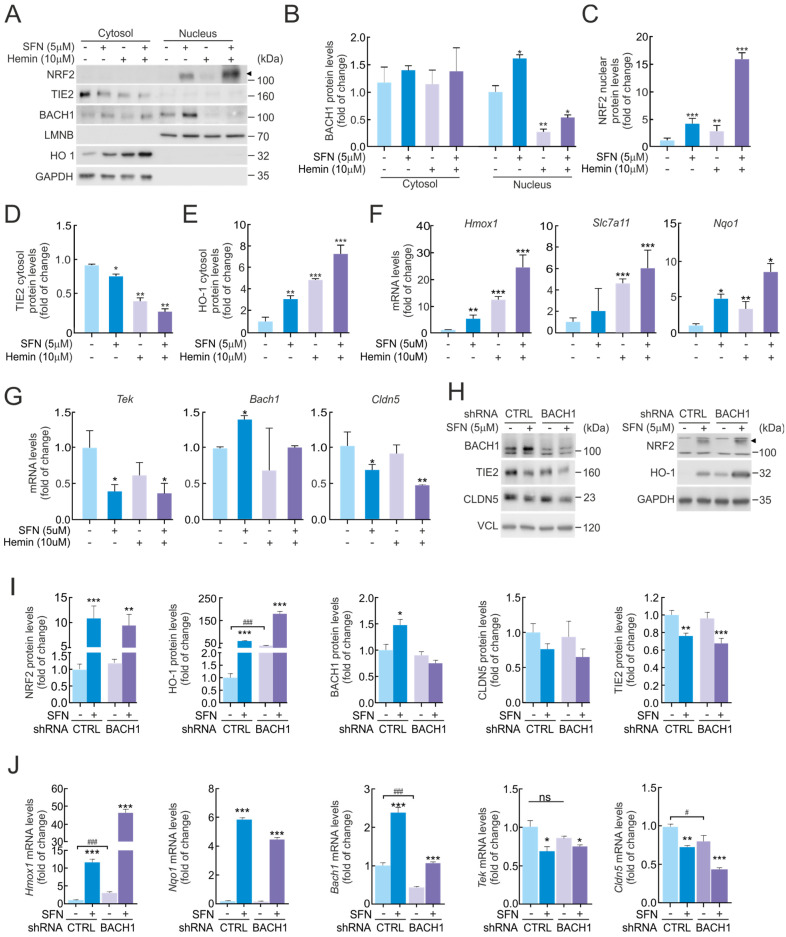
Repression of TIE2/*Tek* levels by NRF2 is not dependent on BACH1. (**A**) Subcellular fraction from bEnd.3 maintained under low-serum conditions (16 h, 1% FBS) and subjected to SFN (5 µM) and hemin (10 µM) treatments for 16 h. Representative immunoblots of NRF2 (arrowhead), BACH1, TIE2, CLDN5, HO-1, and LMNB, and GAPDH as a loading control. Densitometric quantification of BACH1 (**B**), NRF2 nuclear (**C**), TIE2 cytosol (**D**), and HO-1 cytosol (**E**) protein levels from representative immunoblots of A expressed as a ratio of LMNB for nuclear fraction and GAPDH for cytosol fraction, respectively. Data are mean ± S.D. (n = 3). * *p* < 0.05, ** *p* < 0.01 and *** *p* < 0.001 vs. vehicle according to Student’s *t*-test. (**F**,**G**) bEnd.3 cells were maintained under low-serum conditions (16 h, 1% FBS) and subjected to SFN (5 µM) and hemin (10 µM) treatments for 16 h. Transcript levels of *Hmox1*, *Nqo1*, and *Slc7a11* (**F**) and *Tek*, *Bach1*, and *Cldn5* (**G**) were determined by qRT-PCR and normalized to the geometric mean of the levels of *Gapdh*, *Tbp*, and *Actb*. Data are mean ± S.D. (n = 3). * *p* < 0.05, ** *p* < 0.05 and *** *p* < 0.001 vs. vehicle according to Student’s *t*-test. (**H**) bEnd.3 cells were transduced with lentiviral vectors carrying short hairpin RNA against BACH1 (shBACH1) or a scramble sequence (shCTRL). Five days post-transduction, cells were maintained under low-serum conditions (16 h, 1% FBS) and subjected to SFN (5 µM, 16 h). Representative immunoblots of NRF2 (arrowhead), BACH1, TIE2, CLDN5, HO-1, and VCL, and GAPDH as a loading control. (**I**) Densitometric quantification of NRF2, BACH1, HO-1, TIE2, and CLDN5 protein levels from representative immunoblots of H expressed as a ratio of VCL and GAPDH, respectively. Data are mean ± S.D. (n = 3). * *p* < 0.05, ** *p* < 0.01 and *** *p* < 0.001 vs. vehicle, or ### *p* < 0.001 vs. shCTRL according to Student’s *t*-test. (**J**) Transcript levels of *Hmox1*, *Nqo1*, *Bach1*, *Tek*, and *Cldn5* from bEnd.3 cells transduced with shBACH1 or shCTRL maintained under low-serum conditions (16 h, 1% FBS) and subjected to SFN (5 µM, 16 h) were determined by qRT-PCR and normalized to the geometric mean of the levels of *Gapdh*, *Tbp*, and *Actb*. Data are mean ± S.D. (n = 3). * *p* < 0.05, ** *p* < 0.01 and *** *p* < 0.001 vs. vehicle, or # *p* < 0.05 and ### *p* < 0.001 vs. shCTRL according to Student’s *t*-test. ns indicates not significant.

Next, we investigated whether BACH1 could be responsible for the decrease in TIE2/*Tek* expression by employing its classical regulatory compound, hemin [22]. As shown in Figure 3A, bEnd.3 cells were treated with SFN (5 µM), hemin (10 µM), or their combination for 16 h, followed by subcellular fractionation to analyze the distribution of NRF2 and BACH1 between cytosolic and nuclear compartments. As expected, SFN treatment markedly increased NRF2 protein levels, which were detected predominantly in the nuclear fraction (Figure 3A,C), correlating with a robust induction of HO-1 in the cytosol (Figure 3A,E) and a modest reduction in TIE2 protein levels (Figure 3A,D). Consistently, transcriptional analysis confirmed that SFN-induced NRF2 activation upregulated the expression of its canonical target genes *Hmox1*, *Nqo1*, and *Slc7a11* (Figure 3F), while concomitantly reducing *Tek* and *Cldn5* mRNA levels (Figure 3G).

Conversely, hemin treatment markedly reduced BACH1 protein levels in the nuclear fraction by exporting to the cytosol, consistent with the activation of its ubiquitin–proteasome system (UPS)-mediated degradation pathway (Figure 3A,B). However, contrary to expectations, hemin treatment alone did not increase TIE2 protein expression; instead, both hemin and hemin + SFN conditions led to a further reduction in TIE2 levels (Figure 3A,D). These results suggest that the observed effects on TIE2 levels are primarily mediated by NRF2 rather than by BACH1, due to hemin causing oxidative stress. In line with this, BACH1 depletion by hemin did not significantly alter *Tek* or *Cldn5* mRNA levels (Figure 3G), indicating that BACH1 does not exert transcriptional repression on the *Tek* promoter. As positive controls, the mRNA levels of established BACH1 target genes [23]—including *Hmox1*, *Nqo*1, and *Slc7a11*—were strongly upregulated upon hemin treatment (Figure 3F), confirming the expected functional depletion of BACH1.

To confirm whether BACH1 directly contributes to the regulation of TIE2/*Tek* expression—independently of potential off-target effects from hemin—we performed additional experiments using BACH1 genetic ablation. We observed a slight increase in BACH1 expression following SFN (5 µM, 16 h) treatment in shCTRL bEnd.3 cells, both at the mRNA and protein levels. In contrast, no detectable BACH1 expression or significant changes were observed in shBACH1 cells, which was consistent with the absence of effects on TIE2/*Tek* and CLDN5/*Cldn5* levels (Figure 3H–J). Notably, SFN treatment produced a significant reduction in TIE2/*Tek* and CLDN5/*Cldn5* protein and mRNA levels both in shCTRL- and shBACH1-transduced cells (Figure 3H–J).

Overall, these findings indicate that TIE2/*Tek* and CLDN5/*Cldn5* expression are specifically regulated by NRF2 activation in response to SFN and not through BACH1-dependent transcriptional repression mechanisms.

### 2.5. NRF2 Does Not Contribute Directly to TIE2/Tek Expression Levels

Since BACH1 did not appear to mediate the transcriptional repression of TIE2/*Tek*, we next investigated whether NRF2 might regulate *Tek* expression through alternative mechanisms. First, we examined the potential involvement of mRNA stability control in response to SFN, hypothesizing that NRF2 might influence *Tek* expression through miRNA-dependent regulation. Previous studies have shown that miR-144-3p can directly bind the 3′UTR of TIE2/*Tek*, thereby suppressing its expression [24]. Moreover, NRF2 has been reported to transcriptionally activate miR-144-3p by binding to an ARE sequence in its promoter region [25]. Based on this evidence, we sought to determine whether NRF2 could modulate *Tek* mRNA stability through miRNA-dependent mechanisms.

**Figure 4:**
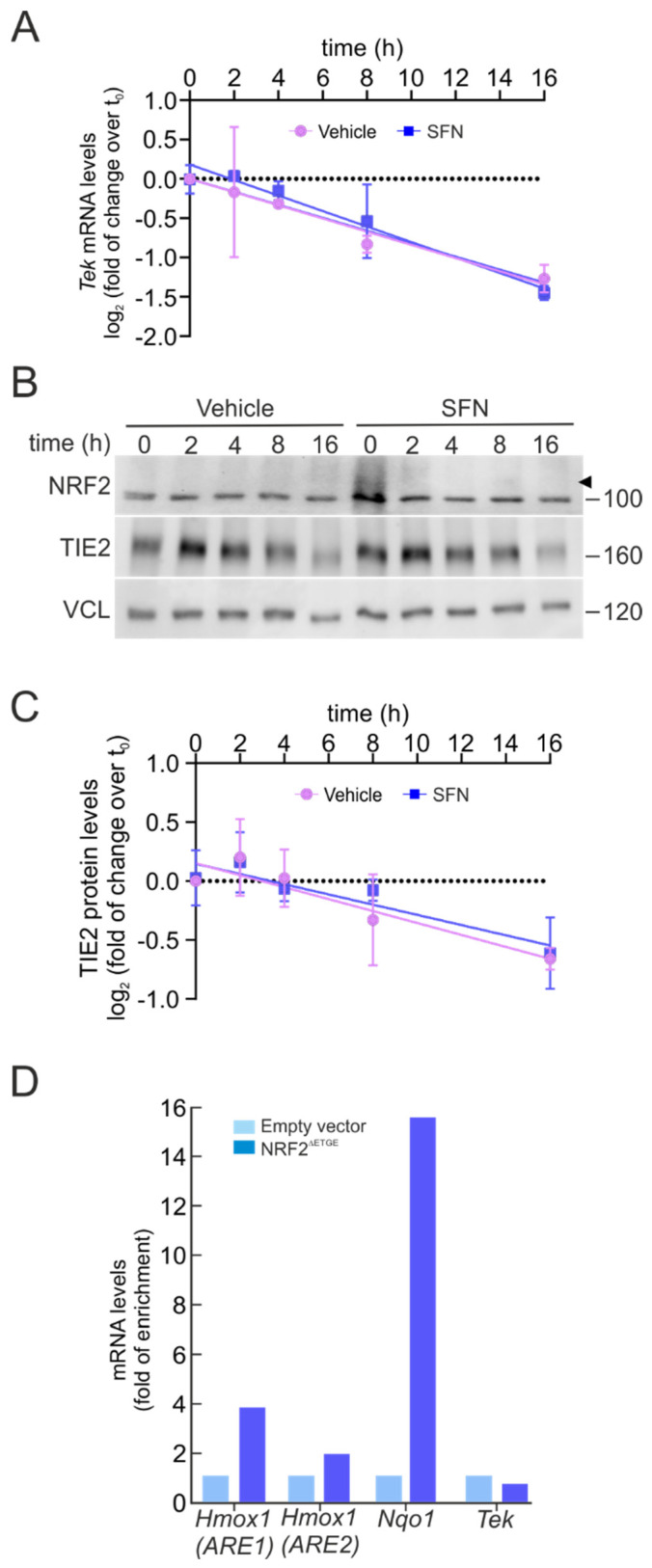
NRF2 does not modify *Tek* mRNA stability and does not bind its promoter. (**A**) bEnd.3 were maintained under low-serum conditions (16 h, 1% FBS), pre-treated with SFN (5 µM, 6 h), and subsequently subjected to actinomycin D (5 µg/mL) or sustained SFN (5 µM) at the indicated time points. The graph depicts the natural logarithm of the relative levels of the *Tek* mRNA as a function of actinomycin D or actinomycin D/SFN incubation time, normalized to the geometric mean of the levels of *Gapdh*, *Tbp*, and *Actb*. The mRNA half-life was determined using the linear part of the degradation curve. Data are mean ± S.D. (n = 3). No statistically significant differences (*p* ≤ 0.05) were detected between vehicle- and SFN-treated groups at any of the analyzed time points according to a two-way ANOVA followed by a Bonferroni post hoc test. (**B**) Representative immunoblots of NRF2 (arrowhead) and TIE2, as well as VCL as a loading control, from bEnd.3 were maintained under low-serum conditions (16 h, 1% FBS), pre-treated with SFN (5 µM, 6 h), and subsequently subjected to CHX (100 µM) or sustained SFN (5 µM) at the indicated time points. (**C**) The graph depicts the natural logarithm of the relative levels of the TIE2 protein as a function of CHX or CHX/SFN incubation time from representative immunoblots of A normalized to VCL. The protein half-life was determined using the linear part of the degradation curve. Data are mean ± S.D. (n = 3). No statistically significant differences (*p* ≤ 0.05) were detected between vehicle- and SFN-treated groups at any of the analyzed time points according to a two-way ANOVA followed by a Bonferroni post hoc test. (**D**) bEnd.3 cells were transduced with lentiviral vectors carrying overexpressed NRF2 insensitive to KEAP1 degradation (NRF2^∆ETGE^) or a lentivirus empty vector. Five days post-transduction, cells were maintained under low-serum conditions (16 h, 1% FBS). Chromatin immunoprecipitation (ChIP) analysis was performed with anti-IgG or anti-PolII antibodies, and the potential AREs in *Tek* with the highest were analyzed by qRT-PCR. The figure shows representative data normalized as the fold of enrichment with the anti-PolII antibody vs. the IgG antibody. The presence of already known AREs in *Hmox1* (ARE1), *Hmox1* (ARE2), and *Nqo1* was analyzed as a positive control, and *Actb* was amplified as a negative control.

The degradation rate of *Tek* mRNA was assessed using an actinomycin D chase assay. Transcription was inhibited by treating bEnd.3 cells with actinomycin D (5 µg/mL) after pre-incubation with SFN (5 µM, 6 h), and *Tek* mRNA levels were subsequently measured by qRT-PCR at various time points. As shown in Figure 4A, the *Tek* mRNA half-life remained unchanged between the vehicle and SFN-treated conditions across all time points.

In parallel, we evaluated the TIE2 protein half-life using the protein synthesis inhibitor cycloheximide (100 µM) under similar pre-treatment conditions. As illustrated in Figure 4B,C, TIE2 protein degradation kinetics were not altered by SFN, displaying a comparable pattern to that observed for *Tek* mRNA stability. These findings suggest that NRF2 does not regulate *Tek* expression through modulation of mRNA or protein half-life, nor through miRNA-dependent mechanisms.

Next, we wondered whether NRF2 could directly repress TIE2/*Tek* transcription. Thus, we searched for potential NRF2-binding sites (AREs) within the *Tek* promoter. Using publicly available ChIP datasets for NRF2 and NRF2-binding partners MAFF and MAFK, as previously described for BACH1, we identified two putative ARE motifs within the TIE2/*Tek* promoter and gene body. The first overlapped with the putative MARE site found previously in the promoter, corresponding to the sequence 5′-CTGACTCAGCT-3′ (chromosomal position: chr4:94738039-94738050; relative score = 0.869). The second was located on intron 8, inside an enhancer element, with the sequence 5′-ATGACTCAGCA-3′ (chromosomal position: chr4:94827452-94827463; relative score = 1).

To experimentally determine whether NRF2 represses *Tek* transcription via this putative ARE sequence, we analyzed RNA polymerase II (Pol II) recruitment, as Pol II occupancy correlates with active transcription [26,27]. ChIP assays were performed using anti-Pol II antibodies in bEnd.3 cells transduced with either an empty vector control (pLENTI-PURO) or a constitutively active NRF2 mutant (NRF2^ΔETGE^) for five days, followed by qPCR with primers targeting the *Tek* (ARE) region and canonical NRF2 target loci (*Hmox1* ARE1/ARE2 and *Nqo1* ARE) as positive controls. As shown in Figure 4D, Pol II occupancy was significantly enriched at the *Hmox1* and *Nqo1* promoters upon NRF2^ΔETGE^ overexpression, confirming NRF2 transcriptional activation. However, no change in Pol II binding was detected at the *Tek* promoter, suggesting that NRF2 does not directly repress TIE2/*Tek* transcription through ARE engagement.

Collectively, these results indicate that NRF2 downregulates TIE2/*Tek* expression through a mechanism that does not involve classical transcriptional repression via BACH1, modulation of mRNA or protein stability, or direct ARE binding to the *Tek* promoter. Rather, our findings suggest that the suppression of TIE2/*Tek* under NRF2 activation likely occurs through indirect regulatory mechanisms, possibly involving secondary transcriptional networks or post-translational signaling events downstream of NRF2 activation.

## 3. Discussion

In this study, we investigated the relationship between NRF2 and one of the BBB components, the TIE2/*Tek* receptor. We found that an increase in NRF2 activity by SFN led to a downregulation of TIE2 levels in both mouse neuroendothelial cell models and in human neuroendothelial cells, as well as in mouse lung endothelial cells, which could be a ubiquitous mechanism in all endothelial-type cells. These results were confirmed throughout bioinformatics and RNA-array analysis, showing that more genes are additionally involved in BBB maintenance. We also found some genes to be upregulated following SFN treatment. These included *Sod1*, a well-known target of NRF2 involved in vasoconstriction and vasodilation [28], as well as *Serpine1*, a regulator that is involved in increasing BBB tightness [29]. Conversely, we detected the downregulation of genes in the NRF2-ablated condition, such as reduced levels of *Pdgfra*, which plays a key role in the function of the BBB, primarily through its involvement in the development and maintenance of pericytes [30]. In addition, *Pf4* and *F2r* are both involved in rapid responses to maintain BBB homeostasis by activating platelet aggregation and thrombus formation, which cover the blood vessel gaps to protect the BBB [31]. Regarding TIE2 signaling, we observed that NRF2 can reduce the expression of some genes belonging to this pathway. These include *Vwf*, which influences the permeability of the BBB and its function by modulating TJ proteins that maintain the integrity of the barrier [32], as well as *Itga5*, a component of the integrin receptor that binds to fibronectin, a key protein in the extracellular matrix [33]. This interaction is vital for the function of endothelial cells, which are the building blocks of the BBB.

In addition, all these BBB-related genes are tightly regulated by NRF2, ruling out possible SFN pleiotropic effects [34] (Figure 1A and Supplementary Figure S2B). SFN exhibits electrophilic properties, which allow it to mediate a wide range of activities resulting from its ability to influence multiple cellular pathways through NRF2-independent mechanisms. To address these concerns, we employed different approaches in this paper, including lentiviral infection overexpressing NRF2^ΔETGE^ and silencing with shNRF2, which allowed us to confirm the results and provide insight into the TIE2/*Tek* inhibition carried out on NRF2-specific targets.

Importantly, our findings do not contradict previous studies describing NRF2-mediated BBB properties [12,35,36,37], as BBB maintenance and angiogenesis are governed by multiple signaling pathways, including sonic hedgehog (Shh) [38], vascular endothelial growth factor [39,40,41], and WNT/β-CATENIN [42]. Notably, NRF2 appears capable of exerting either protective or destabilizing effects on the BBB depending on the cellular and physiological context. Under conditions of moderate or acute stress—such as oxidative injury, inflammation, hyperglycemia, or ischemia–reperfusion—NRF2 activation has repeatedly been shown to enhance BBB integrity through several convergent mechanisms [43]. Recent comprehensive reviews further support that NRF2 promotes TJ protein expression and suppresses matrix metalloproteinases and pro-inflammatory mediators in brain endothelium, reinforcing its classical role in barrier protection [1]. Mechanistically, these observations align with the idea that oxidative stress and inflammation are key drivers of BBB disruption and that NRF2 induction mitigates reactive oxygen species (ROS) accumulation, modulates inflammatory pathways, and preserves the structural and functional stability of the neurovascular unit [1].

Although NRF2 and TIE2 signaling pathways are generally associated with endothelial protection and barrier stabilization, our results reveal that NRF2 activation leads to a consistent but moderate downregulation of TIE2/*Tek* and selected tight junction-related genes. At first glance, this observation may appear counterintuitive. However, several factors related to experimental context and pathway crosstalk may explain these findings. The extent and duration of NRF2 activation are critical determinants of its downstream effects. While acute NRF2 activation under oxidative or inflammatory stress conditions has been widely reported to preserve BBB integrity and promote tight junction stability, sustained or constitutive NRF2 activation—as modeled here by prolonged SFN exposure or NRF2^ΔETGE^ overexpression—may induce adaptive or compensatory responses that attenuate specific angiogenic signaling pathways, including TIE2/*Tek*.

Within the context of the NVU, endothelial–pericyte communication is a fundamental determinant of vascular stability, BBB integrity, and angiogenic remodeling [44]. The ANGPT/TIE2 signaling axis plays a central role in this process by promoting endothelial quiescence, pericyte attachment, and vessel maturation [45]. Reduced endothelial TIE2/*Tek* expression, as observed upon NRF2 activation in our study, could therefore indirectly influence pericyte behavior by altering angiopoietin-mediated paracrine signaling. Consistent with this notion, recent studies have highlighted that disruption of endothelial signaling pathways can impair pericyte coverage and exacerbate vascular permeability and dysfunction [46,47].

Our findings suggest that NRF2 activation may fine-tune endothelial signaling programs that extend beyond endothelial cells themselves. By modulating TIE2/*Tek* levels, NRF2 could influence the balance between vascular stabilization and remodeling, thereby indirectly affecting pericyte recruitment, survival, and contractile function. This mechanism may be particularly relevant under chronic stress or disease conditions, where sustained NRF2 activation could reshape endothelial–pericyte communication and contribute to adaptive or maladaptive vascular responses.

In addition, several considerations suggest that under certain pathological or maladaptive conditions, NRF2 and TIE2 activation may have destabilizing consequences at the neurovascular interface.

First, sustained or constitutive NRF2 activation may shift the redox homeostasis toward a reductive state, thereby impairing the normal ROS-mediated signaling necessary for vascular endothelial adaptation and junctional turnover. The TIE2 receptor and ROS are closely linked, with TIE2 activation by its ligand ANGPT-1 triggering ROS production, which in turn promotes angiogenesis. Specifically, ANGPT-1 activates the TIE2 receptor, which then stimulates ROS generation via enzymes like NADPH oxidase (NOX2 and NOX4) and mitochondria [39,48]. In this way, NRF2 may act as a protective mechanism against excessive ROS by regulating TIE2/*Tek* expression.

Second, in the context of brain tumors or metastases, the TIE2 receptor plays a complex role in cancer by promoting tumor angiogenesis, growth, and metastasis, making it a significant therapeutic target. It is expressed in various cancer cell types and tumor-associated cells, where it signals with its ligands (angiopoietins) to promote blood vessel formation, and can be linked to poor patient prognosis [49,50,51]. Although it has been described that an enhanced NRF2 axis may support abnormal angiogenesis, increased permeability, and survival of aberrant endothelial/vascular cells, moderate NRF2 activation could drive a cytoprotective program against brain tumors or cancer cells by downregulating TIE2/*Tek* expression, thereby preventing the angiogenesis process and metastasis.

Third, dysregulation of NRF2 and/or TIE2 receptors is linked to various chronic pathologies like kidney disease, liver disease, and eye disorders. Under these conditions, factors such as inflammation can reduce protective TIE2 signaling, thereby increasing vascular permeability and contributing to disease progression [52,53,54]. While NRF2 signaling may be impaired or dysregulated, the resulting compensatory overactivation may not restore normal endothelial-barrier function but rather lead to maladaptive responses [55].

We also described additional TJ proteins induced by the TIE2/*Tek* signaling pathway that were not previously identified in bioinformatic and RNA-array analyses. These proteins, which are downregulated by NRF2 activation, include *Cldn5* (CLAUDIN-5), *Cdh5* (CADHERIN-5), *Ocln* (OCCLUDIN), and *Tjp1* (TJ protein 1 or ZO-1). They are key components of the TJs between brain endothelial cells, and likely reflect the hierarchical and modular regulation of BBB junctional complexes [1,56]. Tight junction integrity depends not only on transcriptional control but also on post-translational modifications, protein localization, cytoskeletal anchoring, and paracrine signaling from other components of the neurovascular unit. Therefore, partial transcriptional modulation of individual TJ genes may be sufficient to alter endothelial responsiveness to angiogenic cues without causing overt barrier disruption under basal conditions. Thus, our findings suggest that NRF2 interferes with TIE2/*Tek*-dependent transcriptional programs that maintain TJ protein expression. Through this mechanism, NRF2 activation may negatively affect the molecular architecture and stability of BBB TJ, potentially compromising barrier maintenance and overall endothelial homeostasis.

To investigate how NRF2 could be elicited by its downregulation activity in TIE2/*Tek* expression, we searched for a putative ARE to explain it. It is widely described that NRF2 can activate transcription of its targets through binding to the ARE sequence and stimulating their expression, but its repressor capacity remains unknown. It has been well characterized that NRF2 can reduce and abolish the gene expression of some genes. For instance, it can downregulate MYLK through binding with RPA1 at the NRF2/RPA1 element (NRE) sequence, forming a stable dimer that acts as a transcription factor repressor [17]. Nonetheless, searching the putative NRE sequence in the *Tek* promoter did not reveal an NRF2/RPA1 binding site.

We further explored whether NRF2 could regulate *Tek* expression through mechanisms affecting mRNA stability. Several miRNAs are known to repress TIE2/*Tek* expression, thereby modulating TIE2 signaling and angiogenic responses in various pathological contexts [24,57,58]. Among them, miR-144-3p directly binds the *Tek* 3′UTR and inhibits its expression, and previous studies have shown that NRF2 can activate miR-144-3p transcription by binding to an ARE within its promoter [25]. This raised the hypothesis that NRF2 might downregulate *Tek* expression indirectly through miRNA-mediated destabilization of *Tek* mRNA.

To test this possibility, we assessed *Tek* mRNA stability using an actinomycin D chase assay, which blocks de novo RNA synthesis. As shown in Figure 4C, *Tek* mRNA half-life remained unchanged following SFN treatment, indicating that NRF2 activation does not alter the degradation rate of *Tek* transcripts. Consistently, TIE2 protein turnover exhibited similar kinetics in vehicle- and SFN-treated cells, further demonstrating that NRF2 activation does not affect TIE2/*Tek* expression through miRNA-dependent modulation of mRNA stability. Together, these results exclude the possibility that NRF2 regulates *Tek* expression via microRNA-mediated control of mRNA half-life.

Lastly, to further address this question, we examined whether NRF2 might regulate TIE2/*Tek* expression through its well-known competing partner, BACH1. Under physiological conditions, BACH1 acts as a transcriptional repressor by binding to ARE motifs within antioxidant and detoxification gene promoters, thereby preventing NRF2-driven transcriptional activation [59]. Following SFN treatment, we observed a concomitant increase in both NRF2 and BACH1 at the mRNA and protein levels, raising the possibility that TIE2/*Tek* downregulation could involve BACH1, especially given the presence of a putative MARE site identified in our bioinformatic analysis.

Although NRF2 does not directly bind to the canonical ARE sequence within the BACH1 promoter, NRF2 activation can indirectly influence BACH1 activity through modulation of heme metabolism, particularly via HO-1 induction [60]. Reduced intracellular heme levels diminish heme-dependent BACH1 degradation, thereby stabilizing BACH1. This mechanism makes BACH1 an attractive candidate for mediating the repressive effect on TIE2/*Tek*. However, pharmacological depletion of BACH1 using hemin, as well as genetic ablation through shRNA, failed to produce any detectable changes in TIE2/*Tek* expression (Figure 3). Together, these results indicate that although TIE2/*Tek* levels are clearly dependent on the activation status of NRF2, this repression is not mediated by BACH1, suggesting the involvement of an alternative NRF2-dependent regulatory mechanism.

Study limitations. This study has several limitations that should be acknowledged. First, most experiments were conducted using immortalized endothelial cell lines under in vitro conditions, which, while allowing precise manipulation of NRF2 activity, do not fully recapitulate the cellular complexity and dynamic interactions of the neurovascular unit in vivo. Second, although the consistency of the results across mouse brain, mouse lung, and human endothelial cell lines strengthens the generalizability of our findings, the sample size and experimental scope were necessarily limited to molecular and cellular endpoints, precluding direct assessment of functional BBB properties such as permeability experiments. Finally, despite extensive mechanistic analyses, the precise indirect pathway by which NRF2 downregulates TIE2/*Tek* expression remains unresolved, highlighting the need for future studies employing in vivo models and systems-level approaches to fully elucidate this regulatory axis.

## 4. Materials and Methods

### 4.1. Cell Culture and Reagents

Human coronary microvascular endothelial cells (hCMEC/D3) were grown in endothelial growth medium-2 (EGM-2) (Lonza, Basel, Switzerland). The lung endothelial cells (1g11) were maintained in DMEM (Sigma-Aldrich, St. Louis, MO, USA) supplemented with 20% FBS (HyClone, Logan, UT, USA), 50 U/mL penicillin/streptomycin (15140122, GIBCO, Waltham, MA, USA), 150 μg/mL endothelial cell growth supplement (356006, Corning, NY, USA), 1 mM sodium pyruvate (11360070, GIBCO, Thermo Fisher, Waltham, MA, USA), 100 μg/mL heparin (H3149, Sigma-Aldrich), 1% non-essential amino acids (M7145, Sigma-Aldrich), and 2 mM sodium pyruvate (11360-070, GIBCO). Murine brain microvascular endothelial cell line (bEND.3) was cultured in DMEM supplemented with 10% FBS and 80 mg/mL gentamycin (Laboratorios Normon, Tres Cantos, Madrid, Spain) and supplemented with 1 mM sodium pyruvate, 2 mM glutamine (25030081, GIBCO). Human embryonic kidney 293 with SV40 T antigen (HEK293T) was cultured in DMEM supplemented with 10% FBS and 80 mg/mL gentamycin. All cell cultures were maintained under a controlled atmosphere of 95% relative humidity, 5% CO_2_ concentration, and 37 °C. Other reagents included R,S-sulforaphane (SFN; S8044, LKT Laboratories, Inc., St. Paul, MN, USA), hemin (51280, Sigma-Aldrich, St. Louis, MO, USA), cycloheximide (CHX) (PanReac AppliChem, A0879), and actinomycin D (A9415, Sigma-Aldrich). SFN was diluted in phosphate-buffered salt (PBS), and hemin, cycloheximide, and actinomycin D were diluted in dimethyl sulfoxide.

### 4.2. Plasmids

The vector pcDNA-CMP-ANGPT1 was provided by Dr. Ho Min Kim (Graduate School of Medical Science and Engineering, Korea Advanced Institute of Science and Technology (KAIST), Daejeon, 305-701, Korea).

### 4.3. Production of CMP-ANGPT1 Recombinant

The recombinant protein CMP-ANGPT1 was stably transfected in HEK293 cells using Lipofectamine^®^ 2000 Reagent (116668-019, Invitrogen by Life Technologies, Carlsbad, CA, USA) employing pcDNA-CMP-ANGPT1 (human). Subsequently, the stable clone cells were selected using neomycin antibiotic (G418) at 1 mg/mL. The secreted recombinant proteins in cultured media were purified by column chromatography with streptactin sepharose affinity gel (IBA) and eluted with D-desthiobiotin (Sigma-Aldrich). After purification, the concentration of recombinant proteins was measured by BCA assay and confirmed by Coomassie blue staining of an SDS-polyacrylamide gel (Supplementary Figure S4).

### 4.4. Production of Lentiviral Vectors and Infection

Lentiviruses were produced in HEK293T cells co-transfected with 6 μg of pSPAX2 packaging plasmid (12260, Addgene, Watertown, MA, USA), 6 μg of VSV–G envelope protein plasmid pMD2G (12259, Addgene), and 10 μg of the corresponding lentiviral vector, an empty vector (shCtrl) or with the corresponding shRNA plasmids and packaging vectors, or the pLENTI-PURO empty vector control. Lentiviral vector pLKO.1 shRNA control (shCtrl) (Addgene Plasmid #10879) was purchased from Addgene; pLKO-puro shNRF2 (NM_010902) was purchased from Sigma-Aldrich. Lentiviral plasmid pLENTI-NRF2^∆ETGE^-V5/6xHis-PURO construction from mouse pcDNA3.1-NRF2^∆ETGE^-V5/6xHis is described in [61]. Transfections were performed with Lipofectamine (18324-012, Invitrogen, Waltham, MA, USA) and Plus Reagent (Invitrogen, 11514-015) in Optimem (Invitrogen, 31985-047) for 16 h. Viral supernatants were collected after transfection and filtered through a 0.45 µm membrane. Cells were infected in the presence of 4 μg/mL polybrene (Sigma-Aldrich, ID TR-1003) and selected with 1 μg/mL puromycin (Sigma-Aldrich, ID P8833) for 2–3 days.

### 4.5. In Silico Analysis of TIE2/Tek Putative AREs

TIE2/*Tek* putative MARE/ARE regions from the mouse genome (mm10) were determined using a custom script for the search for transcription binding sites [62]. This script was developed in Python 3.11 and uses a BED file as input, containing merged ChIP-seq peaks for the NRF2 and sMAF (MAFF, MAFK, and MAFG) proteins for ARE sites, or BACH1 and sMAF proteins for MARE sites, retrieved from ChIP-Atlas [63]; a text file containing the list of RefSeq transcripts for TIE2/*Tek* accession numbers; and a position frequency matrix (PFM) file from the JASPAR database containing the corresponding consensus transcription factor binding sites to be computed (MA0150.1 for NRF2 and MA0591.1 for BACH1) [64]. The search was conducted in the 5000 bp upstream of the transcription start site of each transcript and throughout the entirety of the transcript. Only sites with a relative score > 0.85 with respect to the perfect hypothetical MARE/ARE are reported.

### 4.6. Subcellular Fractionation

bEND.3 cells were seeded in p60 plates (5 × 10^5^ cells per plate) and treated with 5 µM SFN or 10 µM hemin for 16 h in serum-free medium. Cytosolic and nuclear fractions were prepared as previously described [65]. Briefly, cells were washed with cold PBS and harvested by centrifugation at 1100 rpm for 10 min. The cell pellet was resuspended in cold buffer A (20 mm HEPES, pH 7.0, 0.15 mm EDTA, 0.015 mm EGTA, 10 mm KCl, 1% Nonidet P-40, 1 mm phenylmethylsulfonyl fluoride, 20 mm NaF, 1 mm sodium pyrophosphate, 1 mm sodium orthovanadate, 1 μg/mL leupeptin) and incubated on ice for 30 min. Then, the homogenate was centrifuged at 2300 rpm for 10 min. The supernatants were kept as the cytosolic fraction. The nuclear pellet was resuspended in cold buffer B (10 mm HEPES, pH 8.0, 0.1 mm EDTA, 0.1 mm NaCl, 25% glycerol, 1 mm phenylmethylsulfonyl fluoride, 20 mm NaF, 1 mm sodium pyrophosphate, 1 mm sodium orthovanadate, 1 μg/mL leupeptin). After centrifugation, the nuclei were resuspended in loading buffer containing 0.5% SDS. The cytosolic and nuclear fractions were resolved in SDS-PAGE and immunoblotted with the indicated antibodies (Supplementary Table S1).

### 4.7. Immunoblotting

Cells were homogenized in lysis buffer (Tris-HCl pH 7.6, 50 mM, 400 mM NaCl, 1 mM EDTA, 1 mM EGTA, and 1% SDS) and the samples were heated at 95 °C for 15 min, sonicated, and pre-cleared by centrifugation. Quantified (23246, Pierce TM BCA Protein Assay Kit, Thermo Fisher, Waltham, MA, USA) proteins were resolved in SDS-PAGE and transferred to Immobilon-P (IPVH00010, Millipore, Burlington, MA, USA) membranes. The proteins of interest were detected with primary antibodies presented in Supplementary Table S1. Membranes were analyzed using the appropriate peroxidase-conjugated secondary antibodies. Proteins were detected by enhanced chemiluminescence (RPN2232, GE Healthcare, Madrid, Spain).

### 4.8. Immunocytofluorescence

bEnd.3 cells were seeded in MW-24 plates (75,000 cells per well), previously incubated and adhered to poly-D-lysine-coated coverslips for one hour, and treated as indicated. Cells were washed with cold PBS and fixed in 4% paraformaldehyde for 15 min at room temperature. After three 5 min washes with PBS, cells were incubated with permeabilization solution (0.25% Nonidet P40 in PBS) for 15 min at room temperature. After briefly washing twice with PBS, the slides were incubated with the indicated primary antibodies (Supplementary Table S1) for 90 min at room temperature in a humidified chamber. Then, cells were washed three times with PBS and incubated with secondary antibodies for 45 min under the same conditions. To visualize the nuclei, cells were stained with DAPI (4,6-diamidino-2-phenylindole). The fluorescence images were captured using appropriate filters in a Leica DMIRE2TCS SP5 confocal microscope (Nussloch, Germany).

### 4.9. Analysis of mRNA Levels

Total RNA extraction, reverse transcription, and quantitative PCR were performed as detailed elsewhere [66]. Primer sequences are shown in the Supplementary Table S2. To ensure that equal amounts of cDNA were added to the PCR, the housekeeping genes *Actb*/*ACTB*, *Tbp*/*TBP*, and *Gapdh*/*GAPDH* were amplified. Data analysis was based on the 2^−ΔΔCT^ method with normalization of the raw data to housekeeping genes (Applied Biosystems, Foster City, CA, USA). All PCRs were performed in quadruplicate. The expression of 84 key genes involved in endothelial function was evaluated with RT^2^ Profiler™ PCR Array Mouse Endothelial Cell Biology (PAMM-015Z, Qiagen, Hilden, Germany) and analyzed with the online GeneGlobe Data Analysis Center (Qiagen).

### 4.10. Chromatin Immunoprecipitation (ChIP) Assay

bEnd.3 cells were grown on 10 cm plates until they reached confluence, subsequently infected with pLENTI-PURO empty vector as control or pLENTI-NRF2^∆ETGE^-V5/6xHis-PURO and selected with 1 μg/mL puromycin for 2–3 days. Briefly, cells were cross-linked with 1% formaldehyde, and the reaction was stopped with 125 mM glycine. Cells were then washed twice with cold PBS, lysed, and sonicated to obtain adequate fragment sizes of DNA. Supernatant was diluted 10-fold with ChIP dilution buffer (0.01% SDS, 1.1% Triton X100, 1.2 mM EDTA, 16.7 mM Tris-HCl, pH 8.1, 167 mM NaCl, 1 mM PMSF, 1 µg/mL leupeptin) and pre-cleared with Protein G Sepharose (17-0618-01, GE Healthcare, Madrid, Spain). Chromatin immunoprecipitation was carried out with anti-RNA Pol II antibody (#sc-17798, Santa Cruz Biotechnology, Dallas, TX, USA) or anti-IgG (ab18413, Abcam, Cambridge, UK). DNA was eluted and purified, analyzing the presence of previously identified putative AREs by quantitative real-time PCR (qRT-PCR) with specific primers (Supplementary Table S3). Samples from at least three independent immunoprecipitations were analyzed.

### 4.11. Image Analysis and Statistics

Unless otherwise indicated, all experiments were performed at least three times, and all data presented in the graphs are the mean of at least three independent samples ± standard error of the mean (SD). Different immunoblot band intensities corresponding to immunoblot detection of protein samples were quantified using Image Lab software (version 6.1) (Bio-Rad, Hercules, CA, USA). Data were analyzed with GraphPad Prism 8.3.0 using an unpaired Student’s *t*-test for comparing two groups, assuming a normal distribution and equal variances. One-way or two-way ANOVA analyses of variance followed by a Bonferroni post hoc test were used for multiple comparisons. A *p*-value ≤ 0.05 was considered statistically significant. Statistically significant differences are indicated in the figures (*** *p* values < 0.001, ** < 0.01 and * < 0.05) (### *p* values < 0.001, ## < 0.01 and # < 0.05).

## 5. Conclusions

In conclusion, we identified an unexpected regulatory role for the transcription factor NRF2, which exerts a repressive effect on the TIE2/*Tek* receptor, a key component in controlling the neurovascular unit and maintaining BBB integrity. The physiological significance of this NRF2-dependent repression will require further investigation in future studies. However, despite extensive analyses, the precise molecular mechanism by which NRF2 downregulates TIE2/*Tek* remains unresolved and warrants deeper exploration.

## Data Availability

The original contributions presented in this study are included in the article/Supplementary Materials. Further inquiries can be directed to the corresponding authors.

## Supplementary Materials

The following are available online at https://www.mdpi.com/article/10.3390/ijms27020770/s1. Refs. [16,67] are cited in Supplementary Materials file.

## Abbreviations

ANGPT1	Angiopoietin1
ARE	Antioxidant response element
BACH1	BTB domain and CNC homolog 1
BBB	Blood–brain barrier
bEnd.3	Brain microvascular endothelial cell line
ChIP	Chromatin immunoprecipitation
CHX	Cycloheximide
CLDN5	Claudin-5
CNS	Central nervous system
DMEM	Dulbecco’s modified Eagle’s medium
ECs	Endothelial cells
EGM-2	Endothelial growth medium-2
FBS	Fetal bovine serum
GAPDH	Glyceraldehyde-3-phosphate dehydrogenase
hCMEC/D3	Human coronary microvascular endothelial cells/D3
HEK293T	Human embryonic kidney 293T
HO-1	Heme oxygenase 1
MARE	MAF recognition element
miR	microRNA
NADPH	Nicotinamide adenine dinucleotide phosphate
NOX	NADPH oxidase
NRE	NRF2/RPA1 element
NRF2	Nuclear factor erythroid 2-related factor 2
NVU	Neurovascular unit
Pol II	RNA polymerase II
qRT-PCR	Quantitative real-time PCR
ROS	Reactive oxygen species
RPA1	Replication protein A1
Shh	Sonic hedgehog
SFN	Sulforaphane
TJ	Tight junction
UTR	Untranslated region
VCL	Vinculin
