# Supplementary material for: Modulation of the Receptor Tyrosine Kinase TIE2/Tek Pathway by NRF2 Activation in Neurovascular Endothelial Cells
Source: Int J Mol Sci. 2026 Jan 13;27(2):770. doi: 10.3390/ijms27020770 (PMC12841328; doi:10.3390/ijms27020770)
Supplement: Supplementary file 1 [file ijms-27-00770-s001.zip › ijms-4089175-supplementary.pdf]

Modulation of the Receptor Tyrosine Kinase TIE2/*Tek* Pathway by NRF2 Activation in Neurovascular Endothelial Cells

Eduardo Cazalla [1,2,3,4,†], Ángel Juan García-Yagüe [1,2,3,4,*,†], Marta Pajares [1,2,3,4], José Jiménez-Villegas [5], Maribel Escoll [1,2,3,4], Ana I. Rojo [1,2,3,4] and Antonio Cuadrado [1,2,3,4,*]

Supplemental Materials

Supplemental Figures

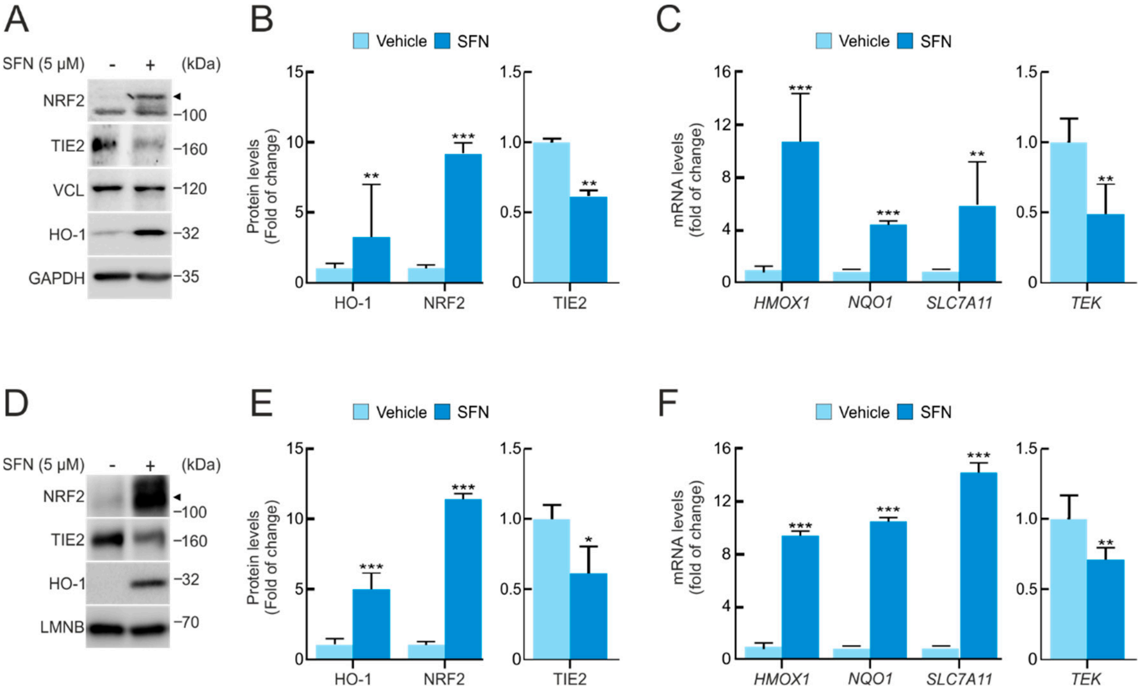

Figure S1. SFN-induced NRF2 activation similarly reduces TIE2/*Tek* levels across multiple endothelial cell types.

(A) Representative immunoblots of NRF2 (arrowhead), TIE2, HO-1, and VCL, and GAPDH as a loading control from human coronary microvascular endothelial cells (hCMEC/D3) maintained under low-serum conditions (16 h, 1% FBS) and treated with SFN (5 µM,16 h). (B) Densitometric quantification of NRF2, HO1, and TIE2 protein levels from representative immunoblots of A expressed as a ratio of VCL and GAPDH, respectively. Data are mean ± S.D. (n = 3). **p < 0.01 and ***p < 0.001 vs. vehicle according to Student´s t-test. (D) hCMEC/D3 cells were maintained under low-serum conditions (16 h, 1% FBS) and subjected to SFN (5 µM,16 h). Transcript levels of

HMOX1, NQO1, SLC7A11, and *TEK* were determined by quantitative real-time PCR (qRT-PCR) and normalized to the geometric mean of the levels of *GAPDH, TBP, and ACTB*. Data are mean ± S.D. (n = 3). **p < 0.01 and ***p < 0.001 vs. vehicle according to Student´s t-test. (D) Representative immunoblots of NRF2 (arrowhead), TIE2, HO-1, and LMNB as a loading control from lung endothelial cells (1g11) maintained under low-serum conditions (16 h, 1% FBS) and treated with SFN (5 µM,16 h). (E) Densitometric quantification of NRF2, HO1, and TIE2 protein levels from representative immunoblots of D normalized with LMNB. Data are mean ± S.D. (n = 3). *p < 0.05 and ***p < 0.001 vs. vehicle according to Student's t-test. (F) 1g11 cells were maintained under low-serum conditions (16 h, 1% FBS) and subjected to SFN (5 µM,16 h). Transcript levels of *Hmox1, Nqo1, Slc7a11,* and *Tek* were determined by quantitative real-time PCR (qRT-PCR) and normalized to the geometric mean of the levels of *Gapdh*, *Tbp,* and *Actb*. Data are mean ± S.D. (n = 3). **p < 0.01 and ***p < 0.001 vs. vehicle according to Student´s t-test.

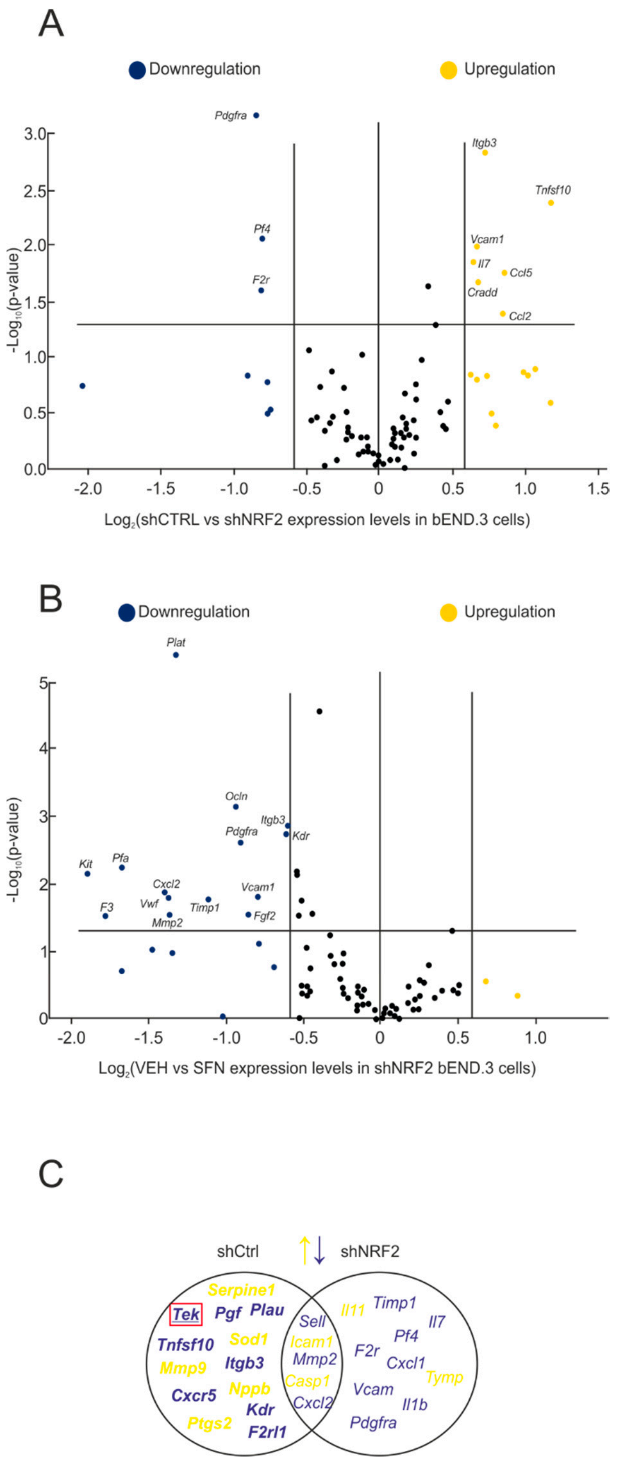

Figure S2. NRF2 modulates endothelial gene expression.

bEnd.3 cells were transduced with lentiviral vectors carrying short hairpin RNA against NRF2 (shNRF2) or a scramble sequence (shCTRL). Five days post-transduction, cells were maintained under low-serum conditions (16 h, 1% FBS) and submitted to SFN (5 µM,16 h). Expression levels of 84 endothelial genes were analyzed by qRT-PCR and normalized to the geometric mean of *Actb*, *Gapdh*, *B2m*, *Gusb* and *Hsp90ab1* levels. (A),

Volcano plot comparing gene expression of shNRF2 versus shCTRL cells. A fold of change greater than 1.5 is represented by yellow (increased expression when compared to shCTRL) and blue (decreased expression when compared to shCTRL) dots. Data are mean of n = 4. Statistical analysis was performed with the GeneGlobe Data Analysis Center from Qiagen. A p-value < than 0.05 was considered significant and is represented by a line. (B) Volcano plot comparing gene expression (expressed as log_{10} of the 2-ΔCt) of vehicle- versus SFN-treated shNRF2 cells. A fold of change greater than 2 is represented by yellow (increased expression when compared to vehicle-treated cells) and blue (decreased expression when compared to vehicle-treated cells) dots. (C) Venn diagram showing gene expression with SFN in shCTRL (left circle) and shNRF2 (right circle) cells. Upregulated and downregulated genes are shown in yellow and blue, respectively. Gene expression changes mediated by SFN-induced NRF2 are depicted in bold.

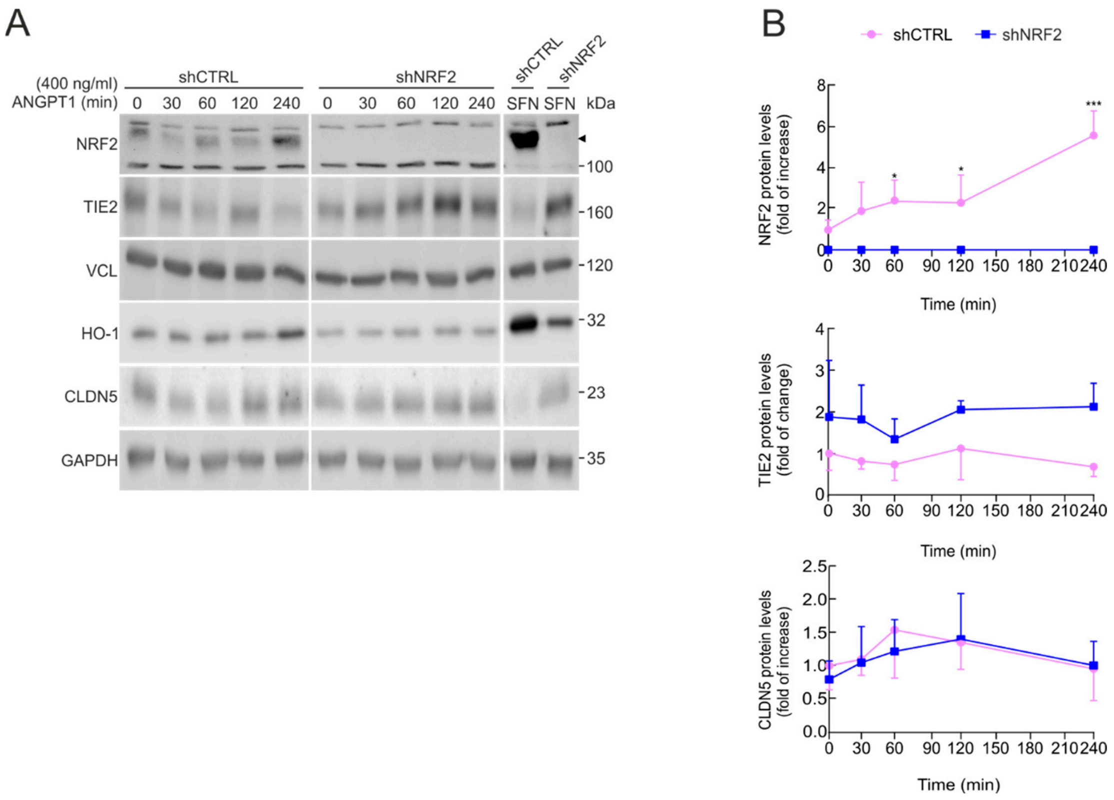

Figure S3. NRF2 genetic ablation does not alter CLDN5 levels by ANGPT1

bEnd.3 cells were transduced with lentiviral vectors carrying short hairpin RNA against NRF2 (shNRF2) or a scramble sequence (shCTRL). Five days post-transduction, cells were maintained under low-serum conditions (16 h, 1% FBS) and subsequently submitted to ANGP1 (400 ng/ml) purified from the supernatant of HEK293T-stable expression (CMP-ANGPT1) at the indicated time points. (A) Representative immunoblots of NRF2 (arrowhead), TIE2, CLDN5, HO-1, and VCL, and GAPDH as a loading control. To check NRF2 silencing, we employed SFN (5 µM,16 h) in both the shRNA condition as a control. (B) Densitometric quantification of NRF2, TIE2, and CLDN5 protein levels from representative immunoblots of A expressed as a ratio of VCL and GAPDH, respectively. Data are mean ± S.D. (n = 3). *$p < 0.05$ and ***$p < 0.001$ vs. time 0 according to a one-way ANOVA with post-hoc Bonferroni's test.

Purification of CMP-ANGPT1 from HEK293 supernatant cells stably transfected

BCA Protein Assay				
Abs	Conc. BSA	Elution CMP-Ang1	Abs	Conc ug/ul
		E0	0,144	0,1320504
0,42	1,6	E1	0,116	-0,0154844
0,277	0,8	E2	0,107	-0,0629063
0,195	0,4	E3	0,108	-0,0576372
0,149	0,2	E4	0,106	-0,0681754
0,123	0			

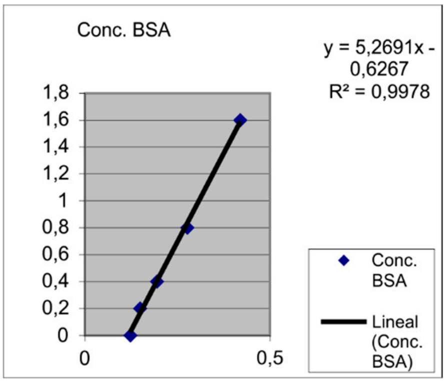

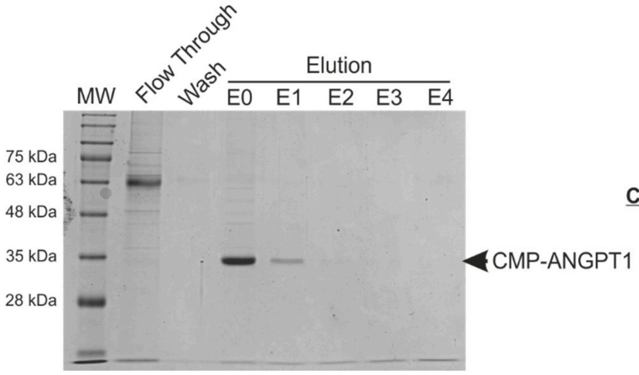

CMP-ANGPT1 estimate concentration 0.13 µg/µl

Figure S4. Quality control of CMP-ANGPT1 production by chromatographic affinity.

CMP-ANGPT1 was prepared as described in the Materials and Methods section. The supernatant from HEK293 cells stably expressing the pcDNA-CMP-ANGPT1 plasmid was collected, and CMP-ANGPT1 was subsequently purified. Its concentration was determined, and the purified protein was resolved by SDS-PAGE and visualized by Coomassie Blue staining.

Supplemental Tables

Table S1. List of antibodies used in this study.

Antibody	Source	Catalog. Number	Dilution (Use)
NRF2	Homemade	(16)	1:5000 (WB)
HO-1	Homemade	(67)	1:2000 (WB)
VCL	Cell Signaling Technology	E1E9V	1:20000 (WB)
GAPDH	Merck-Millipore	CB1001	1:40000 (WB)
TIE2 (mouse)	R&D Systems	AF762-SP	1:2000 (WB)<br>1:200 (IF)
TIE2 (human)	R&D Systems	AF313-SP	1:2000 (WB)
CLDN5	Invitrogen	35-2500	1:1000 (WB)<br>1:150 (IF)
LMNB	Santa Cruz Biotechnology	sc-6217	1:1000 (WB)
BACH1	Santa Cruz Biotechnology	sc-271211	1:1000 (WB)
RNA Pol II	Santa Cruz Biotechnology	sc-17798	1:100 (ChIP)

Table S2. List of primers used for qRT-PCR in this study.

Gene	5´ Forward primer 3´	5´ Reverse primer 3´	Species
HMOX1	TGCTCAACATCCAGCTCTTTGA	GCAGAATCTTGCACTTTGTTGC	Human
SLC7A11	TGCTGGGCTGATTTATCTTCG	GAAAGGGCAACCATGAAGAGG	
NQO1	GTTCATAGGAGAGTTTGCTT	CCTTGCAGAGAGTACATGGA	
TEK	CCTTAGTGACATTCTTCC	GCAAAAATGTCCACCTGG	
TBP	TGCACAGGAGCCAAGAGTGAA	CACATCACAGCTCCCCACCA	
ACTB	TCCTTCCTGGGCATGGAG	AGGAGGAGCAATGATCTTGATCTT	
GAPDH	CTCTCTGCTCCTCCTGTTCGAC	TGAGCGATGTGGCTCGGCT	
Nfe2l2	CAGTCTTCACTGCCCCTCAT	TCTGTCAGTGTGGCTTCTGG	Mouse
Hmox1	CACAGATGGCGTCACTTCGTC	GTGAGGACCCACTGGAGGAG	
Nqo1	GGTAGCGGCTCCATGTACTC	CATCCTTCCAGGATCTGCAT	
Slc7a11	TTCATCCGGCACTATTTTC	CGTCTGAACCACTTGGGTTT	
Tek	GCCGCGGACTGACTACGAGC	GGAGGAGGGAGTCCGATAGACGC	
Cldn5	TTAAGGCACGGGTAGCACTCACG	TTAGACATAGTTCTTCTTGTCGTAATC	
Cdh5	GGACAGCAACTTCACCCTCA	GCAGGTAGTGGAACTTGGCA	
Ocln	ATCCACCTATCACTTCAGA	TAATCTCCCACCATCCTC	
Tjp1	GCCTGCTAAGCCAGTCCATT	CAGTTTCGGGTTTTCCCTTTGA	
Bach1	GCCTGAAGAGGTAACGGTTAAA	GCACACTTCGTCAACATTGTC	
Gapdh	CGACTTCAACAGCAACTCCCACTCTTCC	TGGGTGGTCCAGGGTTTCTTACTCCTT	
Actb	TCCTTCTTGGGTATGGAA	AGGAGGAGCAATGATCTTGATCTT	
Tbp	TGCACAGGAGCCAAGAGTGAA	CACATCACAGCTCCCCACCA	

Table S3. List of primers used for ChIP in this study.

Gene	5´ Forward primer 3´	5´ Reverse primer 3´	Species
Hmox1 (ARE1)	TGACCCGCGTACTTAAAGGG	GGTTCTGCTCGATTCAGGCT	Mouse
Hmox1 (ARE2)	GCCTGAATCGAGCAGAACCA	TAGACTGGGCGGTAACTCGT	
Nqo1	ATATCCTGCTTATGCCCGCC	CCTAGTCCAGCCCCAAACTG	
Tek	GCACCCCAGAGAACAGCTTA	ATTGTGGGGCAGGGGATCT	
Actb	CCTGCAGTGAGGTACTAGCC	CCGCTGTGGCGTCCTATAAA	
